# Is Cannabidiol During Neurodevelopment a Promising Therapy for Schizophrenia and Autism Spectrum Disorders?

**DOI:** 10.3389/fphar.2020.635763

**Published:** 2021-02-04

**Authors:** Cássio Morais Loss, Lucas Teodoro, Gabriela Doná Rodrigues, Lucas Roberto Moreira, Fernanda Fiel Peres, Antonio Waldo Zuardi, José Alexandre Crippa, Jaime Eduardo Cecilio Hallak, Vanessa Costhek Abílio

**Affiliations:** ^1^ Molecular and Behavioral Neuroscience Laboratory, Departamento de Farmacologia, Universidade Federal de São Paulo, São Paulo, Brazil; ^2^ National Institute for Translational Medicine (INCT-TM), National Council for Scientific and Technological Development (CNPq/CAPES/FAPESP), Ribeirão Preto, Brazil; ^3^ Department of Neuroscience and Behavior, Ribeirão Preto Medical School, Universidade de São Paulo, Ribeirão Preto, Brazil

**Keywords:** cannabidiol, Cannabidivarin, schizophrenia, Autism, neurodevelopmental disorders, Prodrome, Prevention, animal models

## Abstract

Schizophrenia and autism spectrum disorders (ASD) are psychiatric neurodevelopmental disorders that cause high levels of functional disabilities. Also, the currently available therapies for these disorders are limited. Therefore, the search for treatments that could be beneficial for the altered course of the neurodevelopment associated with these disorders is paramount. Preclinical and clinical evidence points to cannabidiol (CBD) as a promising strategy. In this review, we discuss clinical and preclinical studies on schizophrenia and ASD investigating the behavioral, molecular, and functional effects of chronic treatment with CBD (and with cannabidivarin for ASD) during neurodevelopment. In summary, the results point to CBD's beneficial potential for the progression of these disorders supporting further investigations to strengthen its use.

## Introduction

Brain development is a critical period for an individual's life; many physiological changes occur during this period, such as neurogenesis and neuronal migration, axonal growth and dendritic maturation, the establishment of nerve cell networks, the formation of new synapses, the proliferation of glial cells, and the myelination (Andersen, 2003). The events and experiences during neurodevelopment will affect the individual's behavioral phenotype and his/her future mental health. It is well established that disturbances occurring throughout critical periods of brain development can disrupt normal brain maturation leading to long-lasting pathological alterations. This highlights the impact of environmental insults on neurodevelopmental psychopathologies such as autism spectrum disorder (ASD) and schizophrenia (Ikonomidou et al., 1999; Kaindl and Ikonomidou, 2007; Dawson et al., 2014; Nicolini and Fahnestock, 2018; Lord et al., 2020). In schizophrenia, a substantial amount of evidence suggests that these disturbances occur during neurodevelopment and are brought about by a combination of genetic and environmental risk factors (Harrison and Weinberger, 2005; Owen et al., 2016; Seshadri et al., 2018). Early periods of brain development are also critical for the establishment of ASD. Even though genetic and epigenetic factors are significant risk factors, environmental events such as gestational and/or perinatal complications could increase the risk of ASD development (Lord et al., 2020). Although the association between neurodevelopmental injuries and neuropsychiatric disorders is not restricted to ASD and schizophrenia, these two disorders share considerable clinical and neurobiological features, ranging from risk factors (e.g., maternal immune activation) to symptoms (such as social disabilities and cognitive deficits) (Boulanger-Bertolus et al., 2018; Barlati et al., 2020). ASD symptoms are frequently observed in patients with schizophrenia and vice versa, with the severity of ASD symptoms being a possible predictor of the severity of schizophrenia symptoms (Barlati et al., 2020).

Furthermore, they also share some pathophysiological mechanisms such as neuroinflammation (Bjorklund et al., 2016; Cattane et al., 2018; Araujo et al., 2019), reduction in thalamus volume, amygdala and thalamus dysfunctions when processing social stimuli (Barlati et al., 2020), as well as glutamatergic, GABAergic (Cattane et al., 2018), and endocannabinoid (ECB) system dysfunctions (Zamberletti et al., 2017; Zador et al., 2019; Borgan et al., 2020; Pietropaolo et al., 2020). The ECB system is widely expressed in the central nervous system, playing roles in synaptic plasticity regulation through retrograde signaling. In a strict sense, it is composed of the cannabinoid receptors type 1 (CB_1_, which is widely expressed in the nervous system) and type 2 (CB_2_, mainly expressed in immune cells), their endocannabinoid signaling molecules (e.g., anandamide (AEA); and 2-arachidonoylglycerol (2-AG)), and their metabolic enzymes (NAPE-PLD, DAGL, FAAH, and MAGL) (Schonhofen et al., 2018).

In this context, the *Cannabis sativa* second-most abundant compound, cannabidiol (CBD), emerges as a potential treatment for these neurodevelopmental psychiatric disorders. CBD is an ECB system modulator that also presents several other mechanisms of action [for detailed information, see Peres et al. (2018b); Schonhofen et al. (2018)]. CBD exerts its effects on both developing and mature brains through several mechanisms, such as modulating the ECB system (either directly via cannabinoid receptors or indirectly by regulating endocannabinoid levels), being an agonist of the vanilloid receptor TRPV_1_, facilitating serotoninergic transmission through 5-HT_1A_ receptors, and interacting with the peroxisome proliferator-activated receptor γ (PPARγ) acting on G-protein-coupled receptor (such as GPR55, GPR3, GPR6, and GPR12) and anti-inflammatory and antioxidant actions.

In this review, we will discuss behavioral and molecular aspects of both clinical and preclinical studies investigating the effects of CBD during neurodevelopment as a potential therapy for ASD and schizophrenia.

### General Aspects of Schizophrenia

Schizophrenia is a psychiatric neurodevelopmental disorder with a lifetime prevalence of just under 1% (Kahn et al., 2015), with the burden of the disease increasing globally (Charlson et al., 2018). It stands out as one of the most debilitating psychiatric disorders because it impairs brain functioning in multiple ways, triggering the expression of positive symptoms (psychosis, characterized by hallucinations, delusions, and disorganized speech), negative symptoms (social dysfunction, avolition, among others), and cognitive symptoms. Negative and cognitive symptoms are more enduring and can precede the first psychotic episode by years, characterizing the prodromal phase (Marenco and Weinberger, 2000; Munro et al., 2002; Schenkel and Silverstein, 2004; Schenkel et al., 2005; Insel, 2010; Larson et al., 2010; Dawson et al., 2014; Millan et al., 2016). More recently, it has been argued that pharmacological interventions during the prodromal phase could delay or even prevent the full-blown manifestation of schizophrenia and preclinical data support this hypothesis (Piras et al., 2014; Gomes et al., 2016; Sommer et al., 2016; Hashimoto, 2019). The establishment of preventive strategies for schizophrenia is essential since the currently available treatment with antipsychotics is most effective for positive symptoms, but ineffective in preventing or slowing schizophrenia progression, besides inducing some serious side effects. On the other hand, there are a significant number of adolescents and young adults presenting reduced social abilities, attenuated psychotic symptoms, and progressive decline in functioning—the so-called individuals at “ultra-high risk” for psychosis—who will not convert to the full-blown manifestation of psychosis (Sommer et al., 2016; Ding et al., 2019). Therefore, potential preventive pharmacological approaches should be beneficial in ameliorating the neurodevelopmental changes associated with schizophrenia. At the same time, they must be safe enough for the approximately 60–70% of at-risk individuals that will not convert to the disorder (Gee and Cannon, 2011; Mokhtari and Rajarethinam, 2013; Piras et al., 2014).

The full comprehension of the mechanisms that underlie schizophrenia progression from the prodromal phase (or earlier) until establishing a psychotic acute state is far from complete. However, at least a portion of these mechanisms have already been elucidated. Impaired functional integration between brain subsystems (e.g., between the hippocampus and the prefrontal cortex (PFC)) and dysfunctions in the organization of brain networks has been suggested to be responsible for the neurocognitive deficits observed in schizophrenia (Peled et al., 2001; Kim et al., 2003; Kim et al., 2005; Meyer-Lindenberg et al., 2005; Benetti et al., 2009; Lee et al., 2012; Dawson et al., 2014; Oh et al., 2017). Neuroinflammation and oxidative stress are also implicated in neurodevelopmental alterations associated with this disorder (Buckley, 2019; Lin and Lane, 2019). Impairments in neurotransmission functions are also described, such as the compromised dopaminergic system in the mesocortical, mesolimbic, and nigrostriatal pathways (Guillin et al., 2007; McCutcheon et al., 2019), the glutamatergic hypofunction in the PFC (Bondi et al., 2012; Snyder and Gao, 2020), and GABAergic, serotoninergic, and ECB system dysfunctions (Eggers, 2013; Schmidt and Mirnics, 2015; Fakhoury, 2017; Cattane et al., 2018; Zador et al., 2019).

Some clinical and preclinical evidence suggests the antipsychotic property of CBD (Zuardi et al., 2012; Saito et al., 2013; Rohleder et al., 2016; Schoevers et al., 2020). Furthermore, CBD does not promote the side effects commonly induced by the traditional antipsychotic drugs (Briles et al., 2012; Leweke et al., 2012; Gomes et al., 2013; Dos-Santos-Pereira et al., 2016; Park et al., 2018). In contrast, the effects that preventive treatments with CBD might have on behavioral and molecular aspects of schizophrenia neuroprogression are still being debated and will be reviewed here.

### General Aspects of Autism Spectrum Disorder

Autism spectrum disorder (ASD) is the fastest-growing neurodevelopmental disorder worldwide, affecting about 1% of the global population and presenting a prevalence four times higher in boys than in girls (Bonnet-Brilhault, 2017; Maenner et al., 2020). According to the DSM-V, ASD core symptoms include impairments in social communication and interaction, restricted or repetitive behaviors, and sensory abnormalities, usually associated with cognitive deficits, intellectual disability, and language delay (American Psychiatric Association, 2013). Also, at least one comorbidity such as epilepsy, gastrointestinal and sleep disorders, and mental health conditions (anxiety, depression, attention-deficit/hyperactivity disorder, and obsessive-compulsive disorder) are present in more than 95% of the patients. At least four comorbidities are associated with ASD in 70% of the cases (Soke et al., 2018). The presence of comorbidities causes a delay in diagnosis, which occurs on average at 4 years old or later (Miodovnik et al., 2015). On the other hand, clinical evidence suggests that the probability of treatment success and the improvement in children’s outcomes increase when interventions occur at very-early ages (2 years old or earlier) (Dawson et al., 2010; Anderson et al., 2014; MacDonald et al., 2014; Rogers et al., 2014; Estes et al., 2015; Pierce et al., 2019).

While improvements in ASD diagnosis have been achieved and cannot be disregarded, early-age diagnostic stability is still not optimal (due to the overlap of clinical symptoms between ASD and other disorders). For this reason, the US Preventive Services Task Force has not yet endorsed early universal screening for ASD (Siu et al., 2016). In contrast, ASD patients still need alternative treatment strategies since current available pharmacological therapies are scarce. Aripiprazole and risperidone (the only FDA-approved drugs for ASD) present limited efficacy besides inducing some side effects such as sedation, increased sleep duration, and weight gain (Tural Hesapcioglu et al., 2020). Therefore, promising therapies should be effective in treating ASD symptoms. Simultaneously, they must be safe enough for both ASD patients and the individuals who will eventually lose their ASD status in a final diagnosis.

The complexity of the pathophysiological mechanisms of ASD is still far from having been fully elucidated. However, knowledge of this topic has advanced considerably, shedding light on important aspects of the disorder. Monogenic mutations with a high risk for the development of ASD partially explain some autistic traits (Shemesh et al., 2016), but a high load of common low-risk variants is also associated with the development of the disorder (Chahrour et al., 2016; Griesi-Oliveira and Sertie, 2017). Moreover, ASD-distinctive genetic architecture produces highly heterogeneous behavioral phenotypes which produces unique symptoms for each patient (Griesi-Oliveira and Sertie, 2017; Lombardo et al., 2019), including some approaches that have classified ASD into subgroups according to the patients’ phenotype (Jacob et al., 2019; Tillmann et al., 2020), while others attempt to classify ASD according to the different patients’ genetic variants (Jeste and Geschwind, 2014). Alterations related to pleiotropic genes associated with ASD can be seen at distinct neurodevelopmental stages (Mitra et al., 2016; Courchesne et al., 2019). During the first and second trimesters of pregnancy, the autistic brain has a high rate of proliferation in the frontal and temporal cortex when compared to neurotypical brains (Courchesne et al., 2007; Courchesne et al., 2011). This leads to irregularities in migration as well as in maturation and differentiation of neurons that result in neural connectivity abnormalities, synaptogenesis damage, and brain overgrowth (Yenkoyan et al., 2017; Courchesne et al., 2019). Local hyperconnections are established in the cortex due to these changes, preventing the functioning of global long-distance connections between brain regions (Courchesne et al., 2007). These cortical changes are accompanied by disruptions in the excitation/inhibitory balance that can cause neuroinflammation and cell death by excitotoxicity (Fang et al., 2014; Courchesne et al., 2019). Other encephalic regions are also disrupted in ASD, including the thalamus and hypothalamus, the amygdala, the striatum, and the hippocampus (Ferhat et al., 2017; Barlati et al., 2020).

At a molecular level, several neurotransmission systems, such as the glutamatergic and the GABAergic (Cattane et al., 2018), are altered in ASD. Similarly, the ECB system (that plays an important role in the modulation of several signaling systems) has also been implicated in the pathophysiology of ASD and has become a target for the development of pharmacological therapies (Wei et al., 2016; Zamberletti et al., 2017; Pietropaolo et al., 2020). Preclinical evidence suggests that its modulation impacts socioemotional reactivity (Servadio et al., 2016; Wei et al., 2016; Folkes et al., 2020), stereotyped behaviors (Servadio et al., 2016; Melancia et al., 2018), learning and memory (Griebel et al., 2015; Qin et al., 2015; Melancia et al., 2018), susceptibility to seizures (Kaplan et al., 2017; Patra et al., 2019; Patra et al., 2020), and regulation of circadian rhythm (Atkinson et al., 2010; Vaughn et al., 2010). All of them are directly or indirectly related to ASD (for detailed review, see Zamberletti et al., 2017).

## Reviewed Studies on Schizophrenia

The terms “cannabidiol” and “schizophrenia” were paired with “neurodevelopment,” “development,” or “preventive” for the search of clinical and preclinical studies in the PubMed database. The inclusion criteria were a) describing the use of CBD-containing products and medications and b) the treatments occurring chronically and during neurodevelopment (from early ages up to late adolescence/beginning of adulthood). Our search yielded only ten results, all on preclinical studies (Table 1). The low number of studies highlights that even though schizophrenia has been recognized for over two decades as a neurodevelopmental disorder (Insel, 2010; Kahn et al., 2015) and that CBD has shown potential antipsychotic properties (Zuardi et al., 2006; Zuardi et al., 2012; Iseger and Bossong, 2015), its use as a potential preventive strategy for at-risk individuals is still poorly explored (Lambert et al., 2016). Four of the studies used a peripubertal/adolescence CBD treatment without continuing it throughout adulthood (Peres et al., 2016a; Peres et al., 2018a; Stark et al., 2019; Stark et al., 2020). In the other six, CBD administration started at late adolescence and extended throughout adulthood (Gomes et al., 2014; Gomes et al., 2015; Osborne et al., 2017; Osborne et al., 2019a; Osborne et al., 2019b; Jimenez Naranjo et al., 2019). Considering the long-term effects of CBD as a preventive strategy, it should be noted that, in four studies (Osborne et al., 2017; Osborne et al., 2019a; Osborne et al., 2019b; Jimenez Naranjo et al., 2019), the chronic preventive effect of CBD could be confounded with a subacute effect (or even an acute effect). In the other two studies, CBD administration occurred concomitantly with the pharmacological induction of the schizophrenic-like phenotype (Gomes et al., 2014; Gomes et al., 2015).

**TABLE 1:** Preclinical results: effects of CBD administration during neurodevelopment on behavioral and molecular evaluations on animal models of schizophrenia.

Species/strain/sex	Model of schizophrenia-like phenotype	Dose and schedule of CBD injections	Measurements	Key behavioral effects	Key molecular effects	Comments	References
Chronic treatment with CBD during peripubertal/adolescence periods							
Rats/SHR/M	Spontaneous SCZ-like phenotype in the SHR strain	0.5, 1, or 5 mg/kg/day (i.p.) from PND30 to PND60	*Behavioral assessment*: catalepsy assessment was performed throughout the period of treatment with CDB, OF, SI, PPI, and CFC, starting on PND90; oral dyskinesia on PND62 and at the end of the other behavioral tasks. *Molecular assessment*: glycemia and serum levels of triglycerides on PND61; quantification of monoamines and their metabolites and the levels of BDNF on PND61 or PND90	0.5 mg/kg CBD prevented the emergence of SHRs’ hyperlocomotor activity and deficits in PPI and CFC	In both strains, 0.5 mg/kg CBD increased the 5-HIAA/serotonin ratio in the PFC on PND61; CBD increased the levels of 5-HIAA in the PFC on PND90	CBD did not induce catalepsy or oral dyskinesia; CBD did not induce metabolic side effects	Peres et al. (2018a)
Rats/SD/M	Single MAM administration (22 mg/kg; i.p) on pregnant dams (GD17); SCZ-like phenotype evaluated in their offspring	10 or 30 mg/kg/day (i.p.) from PND19 to PND39	*Behavioral assessment:* OF, NOR (short-term memory), and SI tasks starting on PND100. *Molecular assessment*: quantification of AEA, 2-AG, PEA, and OEA in the PFC, Hp, and NAc after the last behavioral task; DNA methylation of CNR1 gene promotor and CB1 mRNA and protein expression in the PFC, Hp, and NAc after the last behavioral task	30 mg/kg CBD prevented MAM-induced behavioral alterations in both SI and NOR tasks	30 mg/kg CBD prevented MAM-induced changes in CNR1 promoter DNA methylation, in CB1 mRNA and protein expression in PFC	CBD prevented MAM-induced schizophrenia’s negative- and cognitive-like symptoms in adulthood, without affecting control offspring	Stark et al. (2019)
Rats/SD/M	Single MAM administration (22 mg/kg; i.p) on pregnant dams (GD17); SCZ-like phenotype evaluated in their offspring	30 mg/kg/day (i.p.) from PND19 to PND39	MRI scanning, RT-qPCR, DNA methylation, and molecular modeling of D2 and D3 receptors in complex with CBD and HAL on PND90		30 mg/kg CBD prevented MAM-induced increase in encephalic regional blood flow at the level of the circle of Willis	Computational modeling suggested that CBD could bind preferentially to dopamine D3 receptor than to dopamine D2 receptor	Stark et al. (2020)
Mice/C57Bl/6J/M	Single poly I:C administration (10 mg/kg; i.v.) on pregnant dams (GD9); SCZ-like phenotype evaluated in their offspring	1 mg/kg/day (i.p) from PND30 to PND60	SI and locomotor activity (measured during SI) on PND90	1 mg/kg CBD prevented poly I:C-induced hyperlocomotion		CBD did not alter body weight gain throughout all the experiments	Peres et al. (2016a)

Species/strain/sex	Model of schizophrenia-like phenotype	Dose and schedule of CBD injections	Measurements	Key behavioral effects	Key molecular effects	Comments	References
Rats/SD/M	Single poly I:C administration (4 mg/kg; i.v.) on pregnant dams (GD15); SCZ-like phenotype evaluated in their offspring	10 mg/kg/twice a day (i.p., i.e., 20 mg/kg/day) from PND56 to PND80	NOR (short-term memory), T-maze reward alternation, and SI tasks starting on PND72 and finishing on PND79	10 mg/kg CBD prevented poly I:C-induced deficits in NOR, working memory, and social interaction performance		CBD did not affect total body weight gain, food, and water intake in all experimental groups	Osborne et al. (2017)
Rats/SD/F	Single poly I:C administration (4 mg/kg; i.v.) on pregnant dams (GD15); SCZ-like phenotype evaluated in their offspring	10 mg/kg/twice a day (i.p., i.e., 20 mg/kg/day) from PND56 to PND80	*Behavioral assessment:* NOR (short-term memory), T-maze reward alternation, and SI tasks starting after two weeks of treatment with CBD or vehicle and with a 24 h period interval between tasks. *Molecular assessment:* receptor autoradiography for CB1R, NMDAR, and GABA_A_R binding density assessment in the PFC and Hp measured approximately 10–12 h after the last treatment; FAAH, GluN1, GAD_67_, and PV protein expression in the PFC and Hp measured approximately 10–12 h after the last treatment	10 mg/kg CBD prevented poly I:C-induced deficits in NOR, working memory, and social interaction performance	Poly I:C offspring presented reduced NMDAR binding density in the PFC, while treatment with 10 mg/kg CBD prevented it	CBD increased PV and GAD_67_ expression in Hp, regardless of the gestational manipulation. In control offspring, CBD reduced social interaction, besides NMDAR and CB1R binding density in the PFC	Osborne et al. (2019a)
Rats/SD/M	Single poly I:C administration (4 mg/kg; i.v.) on pregnant dams (GD15); SCZ-like phenotype evaluated in their offspring	10 mg/kg/twice a day (i.p., i.e., 20 mg/kg/day) from PND56 to PND80	Receptor autoradiography for CB1R, NMDAR, and GABA_A_R binding density assessment in the PFC and Hp on PND80; FAAH, GluN1, GAD_67_, and PV protein expression in the PFC and Hp on PND80		Poly I:C offspring presented reduced CB1R binding density in the PFC, while treatment with 10 mg/kg CBD prevented it; poly I:C offspring presented reduced GAD_67_ expression in the Hp, while treatment with 10 mg/kg CBD prevented it	CBD increased GAD_67_ expression in Hp of control offspring; CBD increased PV expression in Hp, regardless of the gestational manipulation	Osborne et al. (2019b)
Rats/SD/M and F	Single poly I:C administration (4 mg/kg; i.v.) on pregnant dams (GD15); SCZ-like phenotype evaluated in their offspring	10 mg/kg/twice a day (i.p., i.e., 20 mg/kg/day) from PND56 to PND80	Receptor autoradiography for M1/M4R binding density assessment in the PFC and Hp on PND80; ChAT and AChE protein expression in the PFC and Hp on PND80		In male offspring, 10 mg/kg CBD treatment attenuated poly I:C-induced changes in M1/M4R binding density in both PFC and Hp (CA1/CA2 and CA3 subregions). In male offspring, 10 mg/kg CBD prevented the poly I:C-induced changes in hippocampal ChAT expression	Neither treatment with poly I:C nor CBD affected the measurements in the female offspring	Jimenez Naranjo et al. (2019)
Mice/C57Bl/6J/M	Daily injections of MK-801 (1 mg/kg; i.p.) for 28 days, starting when animals were 6 weeks old (P1)	15, 30, or 60 mg/kg/day (i.p.) from P6 to P28	*Behavioral assessment:* PPI test on P29. *Molecular assessment:* immediately after PPI, immunohistochemical detection of FosB/ΔFosB and PV and RT-qPCR for GRIN1 gene	30 and 60 mg/kg CBD partially attenuated MK-801-induced impairment in PPI	MK-801 increased FosB/ΔFosB-positive cells in PFC and NAc, while treatment with 60 mg/kg CBD reversed it only in PFC; MK-801 decreased PV-positive cells in PFC, while treatment with 60 mg/kg CBD slightly attenuated it; MK-801 decreased PV-positive cells in PFC and GRIN1 mRNA expression in Hp, while treatment with 60 mg/kg CBD slightly attenuated them	Single CBD injection on P28 did not affect PPI impairments induced by MK-801 injections	Gomes et al. (2014)
Mice/C57Bl/6J/M	Daily injections of MK-801 (1 mg/kg; i.p.) for 28 days (P1–P28), starting when animals were 6 weeks old (P1)	30 or 60 mg/kg (i.p.) from P6 to P28 (i.e., for 23 days)	*Behavioral assessment:* SI and EPM on P29 and NOR (short-term memory) and OF on P30. *Molecular assessment:* immunohistochemical detection of NeuN, GFAP, and Iba1 on P31	CBD (60 mg/kg) attenuated MK-801-induced impairment in SI and NOR	MK-801 increased GFAP-positive cells in PFC, while treatment with CBD (60 mg/kg) slightly attenuated it; MK-801 increased the Iba1-positive cells with a reactive phenotype in PFC and Hp, while treatment with CBD (60 mg/kg) reversed microglial reactivity in all regions		Gomes et al. (2015)

2-Arachidonoylglycerol (2-AG); 5-hydroxyindoleacetic acid (5-HIAA); acetylcholinesterase (AChE); anandamide (AEA); brain-derived neurotrophic factor (BDNF); cannabidiol (CBD); contextual fear conditioning task (CFC); choline acetyltransferase (ChAT); elevated plus maze (EPM); female (F); glutamate decarboxylase 67 kDa isoform (GAD_67_); gestational day (GD); haloperidol (HAL); hippocampus (Hp); high-performance liquid chromatography (HPLC); male (M); methylazoxymethanol acetate (MAM); magnetic resonance imaging (MRI); nucleus accumbens (NAc); novel object recognition task (NOR); N-oleoylethanolamide (OEA); open field behavioral task (OF); N-palmitoylethanolamide (PEA); prefrontal cortex (PFC); offspring’s postnatal day (PND); prepulse inhibition of startle (PPI); parvalbumin (PV); social interaction task (SI); schizophrenia (SCZ); Sprague-Dawley (SD); Spontaneously Hypertensive Rats (SHR).

### Long-Lasting Effects of Cannabidiol Administration as a Preventive Strategy

This section will discuss the long-lasting impact of CBD treatment during earlier periods of development (peripubertal/adolescence) on schizophrenia-like phenotypes in adulthood. Three different schizophrenia animal models were used in these studies: maternal immune activation (MIA) through polyinosinic:polycytidylic acid (poly I:C) administration during the gestational period (Meyer and Feldon, 2012; Haddad et al., 2020), the late gestational antimitotic administration of methylazoxymethanol acetate (MAM) (Lodge and Grace, 2009; Sonnenschein and Grace, 2020), and the spontaneous development of schizophrenia-like behaviors in the Spontaneously Hypertensive Rat (SHR) strain (Calzavara et al., 2009; Calzavara et al., 2011a; Calzavara et al., 2011b; Levin et al., 2011). Chronic administration of CBD during periadolescence presented several benefits regarding the emergence of a schizophrenic-like phenotype in all studies (Peres et al., 2016a; Peres et al., 2018a; Stark et al., 2019; Stark et al., 2020). First, CBD-treated animals showed neither prepulse inhibition of startle deficits (PPI) in the SHR strain model nor spontaneous hyperlocomotion in both the SHR strain and poly I:C models (Peres et al., 2016a; Peres et al., 2018a), behavioral alterations that mimic sensorimotor gating deficits and positive-like symptoms, respectively (van den Buuse, 2010; Almeida et al., 2014; Peres et al., 2016b). Also, cognitive improvements after chronic treatment with CBD were reported for deficits both in the contextual fear conditioning paradigm (CFC, a long-term associative memory task) in the SHR strain model (Peres et al., 2018a) and in the novel object recognition task (NOR, an explicit short-term memory) in the gestational MAM model (Stark et al., 2019). These findings show that the CBD benefits for behaviors that mimic cognitive symptoms are not restricted to a single behavioral phenotype, as they encompass aversive and nonemotional related behaviors, as well as short- and long-term memories. Regarding CBD effects on social interaction impairments, a series of behaviors that mimics the negative symptoms (Almeida et al., 2014; Miyamoto and Nitta, 2014; Wilson and Koenig, 2014), the findings are not consistent. Stark and colleagues (2019) observed improvement in MAM offspring’s social behaviors after CBD treatment, while Peres and colleagues (2018a) did not observe any improvement in the SHR strain’s poor social performance, suggesting that CBD effects on social behaviors can be model-dependent. In parallel, another possible explanation is that CBD effects on social behaviors present a dose-dependent profile since a low range of dosage (0.5, 1, 5, and 10 mg/kg/day) (Peres et al., 2018a; Stark et al., 2019) did not improve social behavior deficits, while a higher dosage (30 mg/kg/day) did (Stark et al., 2019). These results suggest long-lasting beneficial effects of CBD for behaviors that mimic different symptoms of schizophrenia when the treatment occurs during the peripubertal/adolescence period. Clinical and preclinical evidence has already reported that treatment with CBD reduced psychotic symptoms of schizophrenia (Zuardi et al., 2006; Zuardi et al., 2012; Peres et al., 2016b). These studies expand the beneficial effects of CBD, suggesting that it could also be considered as a preventive strategy for at-risk individuals.

Considering the safety requirements of a novel long-term treatment for individuals at risk that will not convert to schizophrenia, potential side effects of prolonged early treatment with CBD were also investigated in these studies. Regarding the positive-, negative-, and cognitive-like behaviors assessed, the authors reported that CBD treatment did not induce any impairment on control animals. In addition, Peres et al. (2018a) observed that chronic CBD treatment did not cause other behavioral alterations (such as catalepsy and oral dyskinesia) or metabolic dysfunctions (such as altered body weight gain, serum levels of glucose, and triglycerides) in both Wistar and SHR strains. Importantly, the absence of behavioral and metabolic dysfunctions following prolonged CBD treatment was observed both immediately and one month after CBD discontinuation. These findings present high translational relevance because CBD showed significant improvements for core schizophrenic-like behaviors without inducing side effects commonly associated with antipsychotic drugs (Muench and Hamer, 2010; Briles et al., 2012; Park et al., 2018). On the other hand, undesired effects of prolonged treatment with CBD have been reported in patients of a wide age range (as reviewed in Schonhofen et al., 2018) and also in mice during peripubertal/adolescence periods (Carvalho et al., 2018a; Carvalho et al., 2018b; Carvalho et al., 2020), highlighting the importance of studies evaluating specifically the potential side effects of chronic treatment with CBD.

Neurochemical alterations following chronic CBD administration were also reported. Stark et al. (2019) investigated the ECB system in the gestational MAM model. They observed increased CB_1_ expression in the PFC as a result of reduced *CNR1* promoter DNA methylation and consequent increase in CB_1_ mRNA expression. These changes were reversed by early chronic treatment with 30 mg/kg/day CBD. The content of the ECB molecules, AEA and 2-AG, and ECB-related molecules, N-palmitoylethanolamide (PEA) and N-oleoylethanolamide (OEA), were also assessed in the PFC. They observed that chronic treatment with CBD increased AEA only in the control offspring and affected 2-AG levels distinctly in control and MAM offspring and that these findings did not directly explain the behavioral alterations. Regarding the dopaminergic neurotransmission, Stark et al. (2020) observed an increased D_2_ mRNA content in PFC of MAM offspring that was not affected by chronic treatment with CBD. Intriguingly, alterations in D_2_ mRNA content did not reflect changes either in D_2_ protein expression or in DNA methylation of D_2_ gene regulatory regions that were not affected by the MAM insult or CBD treatment. They also found that D_3_ mRNA content was increased in PFC, hippocampus, and NAc of the MAM offspring, while treatment with CBD reduced it in all three regions without altering it in control offspring. In fact, D_3_ mRNA content was almost absent in PFC and NAc of the MAM offspring treated with CBD. However, similar to D_2_ results, D_3_ mRNA content alterations did not reflect DNA methylation changes of D_3_ gene regulatory regions while D_3_ protein expression was not evaluated. An absence of effect of CBD on the dopaminergic system was reported by Peres and colleagues (2018a): the early long-term treatment did not change the increased dopamine levels in PFC of the SHR strain at 90th postnatal day with CBD (lower doses than the 30 mg/kg/day used in the study by Stark et al., 2020). Additionally, Stark et al. (2020)—using molecular modeling approaches—proposed that CBD may act as a weak partial agonist of D_3_ receptors once it can favorably bind to dopamine D_3_ rather than to dopamine D_2_ receptors. This finding is in accordance with a previous study that computationally predicted the D_3_ receptor as a potential target for CBD (Bian et al., 2019). Nevertheless, D_2_ receptors cannot be disregarded as a potential target for CBD, since CBD has also been proposed to act as a partial agonist of these receptors, similarly to the antipsychotic aripiprazole (Seeman, 2016).

Besides the CBD effects on ECB and dopaminergic systems discussed above, chronic CBD treatment effects on the serotoninergic system and the brain-derived neurotrophic factor (BDNF) were also reported for the SHR strain model (Peres et al., 2018a). The authors found that the SHR strain presents reduced levels of serotonin in PFC at the 61st but not at the 90th postnatal day and that chronic CBD treatment was not able to recover it. On the other hand, increased levels of the serotonin metabolite 5-hydroxyindoleacetic acid (5-HIAA) were observed in the PFC of both Wistar- and SHR-treated animals one month after CBD discontinuation. In the same direction, the 5-HIAA/serotonin ratio was also increased one day after CBD administration ceased, although a more pronounced effect was observed in the SHR strain. Regarding BDNF levels, no CBD effects were reported. These data suggest that chronic treatment with CBD during peripubertal/adolescence periods increases serotonin turnover in the PFC and supports the role of the serotoninergic system in the CBD effects on the brain (Russo et al., 2005; Linge et al., 2016).

Finally, neuroanatomical and functional alterations were also evaluated (Stark et al., 2020). An elevated regional cerebral blood flow (CBF) in the circle of Willis and a regional CBF reduction in the hippocampus were observed in the MAM offspring, following other clinical and preclinical studies showing altered CBF in schizophrenia (Goozee et al., 2014; Drazanova et al., 2018; Drazanova et al., 2019). Chronic treatment with CBD reversed the changes in the circle of Willis but not in the hippocampus (Stark et al., 2020). Moreover, CBD reduced regional CBF in the somatosensory cortex of MAM offspring but not of control offspring. No alterations were observed in relation to PFC and NAc. In parallel, the enlargement of lateral ventricles—a structural alteration commonly observed in both patients and animal models of schizophrenia (Le Pen et al., 2006; Kempton et al., 2010)—in the MAM offspring was not prevented by the long-term treatment with CBD (Stark et al., 2020). Interestingly, although the authors have not discussed the possible relationship between the CBF and the anatomical changes, the enlargement of lateral ventricles could be a consequence of the reduced hippocampal blood flow resulting in a reduction of the hippocampal volume, as observed by Stark et al. (2020) and by others that also used the gestational MAM model to investigate this issue (Le Pen et al., 2006). Even though this topic needs to be further explored, it seems that neither chronic treatment with CBD nor chronic treatment with an antipsychotic drug (haloperidol) can reverse these neuroanatomical and functional alterations (Stark et al., 2020).

Despite the limited number of studies investigating the effects of CBD treatments during an early prodromal-like phase of schizophrenia (so far, only three studies investigated its impact on animals’ behavior), the results pointing out the benefits for its use are quite robust and promising. Nevertheless, it remains unclear whether CBD administration is hindering the emergence of schizophrenia-like behaviors or reversing the early signs already present in a prodromal phase. Some aspects of schizophrenia-like behaviors in those animal models were previously described and speculations can be inferred from them. In the SHR strain, social impairments and CFC deficits have already emerged during puberty/adolescence, while spontaneous hyperlocomotion and PPI deficits appear only during adulthood (Niigaki et al., 2019). Similar results about the early emergence of social impairments and the late emergence of hyperlocomotion were observed in other animal models, including the gestational MAM model (Sams-Dodd et al., 1997; Le Pen et al., 2006). Also, the early emergence of cognitive deficits (Su et al., 2014; Latusz et al., 2017) and the late emergence of PPI deficits were also reported in other animal models, including the MIA (through poly I:C administration) and the gestational MAM models (Le Pen et al., 2006; Ozawa et al., 2006; Uehara et al., 2010; Latusz et al., 2017; Takahashi et al., 2019). These preclinical results are in agreement with the course of schizophrenia: the early appearance of negative- and cognitive-like symptoms (i.e., a prodromal phase) followed by a later emergence of sensorimotor gating deficits and positive-like symptoms (Marenco and Weinberger, 2000; Larson et al., 2010; Millan et al., 2016). Based on the above-discussed reports, even though there are some conflicting results about the timing in which the emergence of the behavioral alterations occurs (Le Pen et al., 2006; Takahashi et al., 2019), it can be speculated that early chronic treatment with CBD during peripubertal/adolescence may be able to recover the already established behavioral deficits and/or prevent the emergence of the late abnormalities observed in schizophrenic-like models. Notably, CBD effects last more than a month after the treatment was discontinued, suggesting that prolonged treatment with CBD during a “prodromal phase” induced long-lasting brain changes that altered the course of the pathophysiological mechanisms underlying schizophrenia, delaying the progression of the disorder.

### Effects of Prolonged Cannabidiol Administration During Later Periods of Development on the Schizophrenia-Like Phenotype

This section will discuss CBD treatment's impact during later periods of development (end of adolescence/early adulthood) on the schizophrenia-like phenotype in adulthood. Two different schizophrenia animal models were used: the already mentioned MIA through poly I:C administration during the gestational period (Osborne et al., 2017; Osborne et al., 2019a; Osborne et al., 2019b; Jimenez Naranjo et al., 2019) and a late adolescence/early adulthood transient NMDA receptor antagonism model (Li et al., 2011; Uttl et al., 2018; Ma et al., 2020) through daily MK-801 administration during 28 days (Gomes et al., 2014; Gomes et al., 2015). Similar to the above-discussed data, prolonged administration of CBD during late adolescence/early adulthood also presented several benefits regarding the manifestation of a schizophrenia-like phenotype in all the studies. Osborne and colleagues (2017, 2019a) showed that MIA through poly I:C administration in the dams induced social impairments and cognitive deficits in male and female offspring. Interestingly, working memory deficits in the “rewarded T-maze test” at early adulthood were sex-dependent, being observed only in male offspring. Short-term explicit memory impairment in the NOR task was observed in both male and female offspring, suggesting that different cognitive processes are affected in distinct ways in this model. Regardless of the sex, prolonged treatment with CDB (10 mg/kg twice a day, i.e., 20 mg/kg/day) from PND56 to PND80 attenuated all the behavioral impairments evaluated. In contrast, control females treated with CBD presented a reduction in social interaction that was not observed in male ones. Although this result indicates a putative sex-specific side effect of CBD in healthy individuals, this study's experimental design does not allow identifying if this alteration is a consequence of chronic or acute CBD administration. Moreover, from the ten studies included in this review (Table 1), only one of them evaluated behavioral alterations in females, challenging the discussion of a possible sex-dependent effect of CBD.

Effects of prolonged treatment with CBD on social performance and short-term explicit memory impairments were also evaluated in the transient NMDA receptor antagonism model through chronic MK-801 administration at late adolescence/early adulthood (Gomes et al., 2015). The authors found that treating the animals for 23 days (starting on the sixth day after the first MK-801 administration) with 60 mg/kg/day CBD, but not 30 mg/kg/day, attenuated negative- and cognitive-like symptoms (in the social interaction test and the NOR, respectively). They also found that neither the late chronic MK-801 administration nor the late prolonged treatment with CBD induced changes in locomotor behaviors (in the OF task) and anxiety-like behaviors (in the EPM task), which are in accordance with some other reports (Li et al., 2011; Schiavon et al., 2016; Uttl et al., 2018) but not with others (ElBatsh et al., 2012; Uttl et al., 2018) that investigated their effects in similar age periods. Although further investigation is needed, the use of distinct species/strains and protocols to investigate CBD or MK-801 effects in these studies can account for the different outcomes (Viola and Loss, 2014; Uttl et al., 2018). In another study, Gomes and colleagues (2014) investigated the effects of the same prolonged treatment with CBD on sensorimotor gating deficits induced by the same protocol (chronic MK-801 administration at late adolescence). Their results suggest that prolonged treatment with 60 mg/kg/day CBD produced only a slight attenuation of PPI impairments.

These studies follow the data discussed in the previous topic, giving further support for the beneficial effects of CBD even when its administration occurs during late periods of neurodevelopment. Nevertheless, it should be noted that some of the results are conflicting (e.g., the effects of prolonged treatment with CBD on anxiety-like behaviors) and that the data are scarce (so far, only four studies investigated the effects of prolonged treatment with CBD during late adolescence/early adulthood on schizophrenia-like behaviors).

Side effects of prolonged treatment with CBD were poorly explored in the above-mentioned studies. Osborne and colleagues' (2017, 2019a) findings suggest a sex-dependent effect of poly I:C treatment on body weight and water intake but not on food intake. Poly I:C female offspring seem to be heavier and consume more water at adulthood than the control female offspring. No differences in these variables were observed in male subjects. Regarding the transient NMDA receptor antagonism model, no conclusions can be drawn about the influence of sex on these variables, since only males were used in these studies (Gomes et al., 2014; Gomes et al., 2015). Similar to the poly I:C model, MK-801 male subjects did not present differences in body weight when compared to control subjects. Regardless of the sex and the schizophrenia-like model, prolonged treatment with CBD did not induce any alteration in these variables. Therefore, besides the above-discussed decreased social interaction observed in females, no other adverse effects of prolonged treatment with CBD during late development periods were reported in these studies. However, some studies observed the emergence of adverse effects after repeated CBD administration in similar age periods, such as increased anxiety-like behaviors (ElBatsh et al., 2012) and decreased neurogenesis (Schiavon et al., 2016), highlighting the fact that further confirmatory studies are needed.

Molecular and functional alterations in the brain following prolonged treatment with CBD were also reported. Regarding the ECB system, Osborne and colleagues (2019a, 2019b) observed that in the poly I:C model CB_1_ binding density was affected in a sex-dependent way. While CB_1_ binding density was decreased in the PFC of poly I:C male offspring, it was not altered in female ones. The prolonged treatment with CBD reversed the changes in male offspring (Osborne et al., 2019b). In addition, it decreased CB_1_ binding density in the control female offspring (Osborne et al., 2019a).

Regarding FAAH expression, it was not affected either in the poly I:C and control offspring, independently of the sex and of the treatment with CBD (Osborne et al., 2019a; Osborne et al., 2019b). In contrast to the decreased CB_1_ binding density found in the above-mentioned study, the previously discussed study by Stark et al. (2019) found an increased CB_1_ expression in MAM male offspring. Moreover, early chronic treatment with CBD (in a different dose and developmental period) in MAM male offspring reversed this change by reducing CB_1_ expression to control levels (Stark et al., 2019) while in the study by Osborne et al. (2019b) the late prolonged treatment with CBD in poly I:C male offspring normalized CB_1_ binding density by increasing it to control levels. Together, these results suggest that the CB_1_ receptor is affected distinctly in the different models and by the different protocols of CBD administration.

Sex-dependent results were also found for the glutamatergic system, in which the poly I:C model decreased NMDA receptor binding density in the PFC of female offspring (Osborne et al., 2019a), but not of male ones (Osborne et al., 2019b). Interestingly, expression of the obligatory GluN1 subunit was unaffected in either the poly I:C and control offspring, independently of the region analyzed (PFC or hippocampus), and the sex and the treatment with CBD (Osborne et al., 2019a; Osborne et al., 2019b), suggesting that gestational poly I:C injection is affecting the functionality of the glutamatergic system (glutamate synthesis, release, or reuptake, for instance, or even the composition of NMDA receptor) without necessarily interfering in the amount of NMDA receptor expressed. Prolonged treatment with CDB (10 mg/kg twice a day; i.e., 20 mg/kg/day) from PND56 to PND80 effectively reverted the decreased NMDA receptor binding density in the poly I:C female offspring. In contrast, in control female offspring, it decreased NMDA receptor binding density in the PFC similarly to gestational injection of poly I:C (Osborne et al., 2019a). These data are not in accordance with Gomes and colleagues’ study (2014) that showed no alteration in *GRIN1* mRNA expression in the PFC and striatum of male mice subjected to chronic MK-801 administration (daily injections for 28 days) at late adolescence/early adulthood but did show a decrease in the hippocampus. This change was slightly attenuated when prolonged treatment with 60 mg/kg/day CBD occurred concomitantly (for 23 days) with MK-801 administrations.

Regarding the GABAergic system, Osborne et al. (2019a); Osborne et al. (2019b) reported that prolonged treatment with CBD increased parvalbumin (PV) expression in the hippocampus (but not in the PFC) regardless of the gestational manipulation or the sex of the offspring, while gestational poly I:C injection did not induce any alteration *per se*. On the other hand, Gomes et al*.* reported a decreased number of PV-positive cells in the PFC (but not in the striatum or the hippocampus) of male mice subjected to chronic injections of MK-801 during late adolescence/early adulthood (Gomes et al., 2014). This alteration was slightly attenuated when CBD was concomitantly administrated. It is important to note that these results are not necessarily conflicting, because the expression of PV can be altered without affecting the number of PV-positive cells and vice versa. Sex-dependent effects were reported for GAD_67_ expression (Osborne et al., 2019a; Osborne et al., 2019b). Gestational poly I:C injection decreased GAD_67_ expression in the hippocampus of male offspring but not female ones. Prolonged treatment with CBD increased hippocampal expression of GAD_67_ regardless of the sex or gestational manipulation, bringing it back to control levels in male offspring while increasing it above control levels in female ones. No alterations were observed regarding GABA_A_ receptor binding density (Osborne et al., 2019a; Osborne et al., 2019b).

The effects of prolonged treatment with CBD on the cholinergic system were also investigated. Jimenez Naranjo et al. (2019) results showed that gestational poly I:C administration reduced muscarinic M1/M4 receptors binding density in the PFC and hippocampus of male offspring, while the prolonged treatment with CDB (10 mg/kg twice a day; i.e., 20 mg/kg/day) from PND56 to PND80 slightly attenuated this alteration in the poly I:C male offspring. On the other hand, this treatment with CBD reduced muscarinic M1/M4 receptors binding density in the control male offspring at similar levels of the poly I:C ones. There was no evidence of M1/M4 receptors binding density alterations induced by either the gestational poly I:C administration or the postnatal treatment with CBD in female offspring. The authors also reported that gestational poly I:C administration reduced hippocampal choline acetyltransferase (ChAT) expression of male offspring, but not female ones, while acetylcholinesterase (AChE) protein expression was not altered in either sex. Prolonged treatment with CBD did not affect these proteins in both male and female offspring.

To investigate putative functional effects of prolonged treatment with CBD on chronic administration of MK-801 at the late adolescence/early adulthood model, Gomes et al. (2014) also evaluated the FosB/ΔFosB expression (an indication of sustained neuronal activation) (Nestler et al., 1999). The authors reported an increased number of FosB/ΔFosB-positive cells in PFC and NAc (but not in dorsal striatum and hippocampus) after chronic MK-801 injection. Concomitant administration of CBD was able to revert this increase in the PFC but failed to alter it in the NAc. On the other hand, CBD treatment did not change the number of FosB/ΔFosB-positive cells in control animals.

Finally, only one study investigated the effects of prolonged treatment with CBD on neuroinflammation. Gomes and colleagues (2015) reported astrogliosis in the PFC of chronic MK-801-treated animals in late adolescence/early adulthood. Microglial reactivity was also observed in both the PFC and the hippocampus of these animals. Concomitant administration of CBD for 23 days attenuated the astrogliosis induced by MK-801 in the PFC. Furthermore, prolonged CBD treatment was also capable of reverting microglial reactivity in both the PFC and hippocampus of these animals. Prolonged treatment with CBD did not induce any glial changes in control animals. These results confirm the already described anti-inflammatory effects of CBD (Burstein, 2015).

Although the results of the studies employing the poly I:C model are interesting (Osborne et al., 2017; Osborne et al., 2019a; Osborne et al., 2019b; Jimenez Naranjo et al., 2019), yielding sex-dependent differences in the schizophrenia-like phenotype, which are in accordance with the course of the disorder in humans (Abel et al., 2010; Ochoa et al., 2012; Barajas et al., 2015), these studies performed behavioral and neurochemical evaluations while the treatment with CBD was still ongoing. CBD's long-term effects can only be speculated as we cannot distinguish them from its acute effect. In parallel, the studies employing a blockade of NMDA receptors at late adolescence/early adulthood (Gomes et al., 2014; Gomes et al., 2015) performed the CBD treatment concomitantly to the MK-801 administration (starting on the sixth day after the beginning of MK-801 injections). Recently, a study from the same group (Rodrigues da Silva et al., 2020) showed that MK-801 administrations twice a day (in the dose range of up to 2 mg/kg/day) for seven consecutive days were not enough to induce schizophrenia-like behavioral alterations (measured eight days after the last MK-801 injection, i.e., on the 15th day of the experiment). In contrast, MK-801 injections twice a day (0.5 mg/kg, i.e., 1 mg/kg/day) for fourteen consecutive days induced social impairments and cognitive deficits (in the social interaction test and in the NOR, respectively, which were measured at both one and eight days after the last MK-801 injection, i.e., on the 15th and 22nd days of the experiment). Thus, in the two studies by Gomes et al. (2014), Gomes et al. (2015), CBD's effects on the development and progression of the behavioral and neurochemical changes cannot be distinguished from the action of CBD directly interfering with MK-801 mechanisms of action. On the other hand, it should be noted that the subacute treatment with CBD was effective in reversing the NMDA receptor antagonism-induced behavioral changes even after MK-801 injections were suspended (Rodrigues da Silva et al., 2020).

Clinical evaluations of the effects that a long-term CBD treatment might have on the course of the neurodevelopmental pathophysiological mechanisms associated with the emergence of schizophrenia are still lacking. Notwithstanding, beneficial effects of acute or subacute treatments with CBD for individuals at clinical high risk for psychosis (CHR, at late adolescence/early adulthood) have been recently described. Functional magnetic resonance imaging studies have shown that individuals at clinical high risk for psychosis (CHR, aging from 18 to 35 years) present altered activation of some brain regions—such as the striatum and the medial temporal cortex—during cognitive and emotional processing. Although the direction of changes in these regions may vary according to the task, the administration of a single dose of CBD (600 mg) promotes a normalization of the dysfunction observed (Bhattacharyya et al., 2018; Davies et al., 2020). In addition, the insular dysfunction presented by CHR subjects during motivational salience processing is also attenuated by this same single dose of CBD (Wilson et al., 2019). Adding to the beneficial effects of CBD on abnormal brain activities, another study of the same group reported that a seven-day treatment with CBD (600 mg/kg) partially attenuated abnormal cortisol levels and anxiety and stress perception induced by social stress in CHR individuals (Appiah-Kusi et al., 2020).

## Reviewed Studies on Autism Spectrum Disorder

Here, we reviewed the impact that treatment with CBD during neurodevelopment has on behavioral and molecular aspects of ASD. Firstly, the term “cannabidiol” was paired with “autism” or “autism spectrum disorder” for the search of clinical and preclinical studies in the PubMed database. Additional searches were carried out in the reference list of the studies found in the first search. Since no preclinical studies were found, we expanded the search using the term “cannabidivarin” (CBDV, a propyl analog of CBD) as an alternative phytocannabinoid molecule for CBD. The final inclusion criteria were a) describing the use of products and medications containing CBD or CBDV in the treatment of ASD and b) the treatments occurring chronically and during the neurodevelopment (from early ages up to late adolescence/beginning of adulthood). Only five studies were included: four clinical trials using cannabis oil extract and one preclinical study using CBDV (Table 2). Case reports were not included.

**TABLE 2:** Clinical and preclinical results: effects of CBD administration during neurodevelopment on behavioral and molecular evaluations in both animal models and patients of autism spectrum disorders.

Sex/age	Study design	Dose and schedule of CBD administration	Measurements	Main results	Comments	References
Clinical studies						
*N* = 60 (83% M)/5–18 years old (mean 11.8 ± 3.5)	Retrospective study; children with ASD and refractory disruptive behaviors investigated after 7–13 months of treatment	CBD/THC ratio of 20:1 oil (SL), 2–3 times a day with doses up-titrated over 2–4 weeks (starting CBD dose was 1 mg/kg/day; maximal CBD dose was 10 mg/kg/day). The mean total daily dose was 3.8 ± 2.6 mg/kg/day CBD and 0.29 ± 0.22 mg/kg/day THC for children who received three daily doses (*n* = 44) and 1.8 ± 1.6 mg/kg/day CBD and 0.22 ± 0.14 mg/kg/day THC for children who received two daily doses (*n* = 16)	CGIC; HSQ-ASD; APSI; retention rates; modified Liverpool adverse events profile	All had severe behavioral problems based on CGI-S (scores of 6 or 7); 29 patients with insufficient response used cannabis strains with lower CBD: THC ratios (6:1; maximal CBD dose was 5 mg/kg/day); retention rate of 73% (mean treatment duration: 10.9 ± 2.3 months); improvement in CGIC: 61% in behavioral outbreaks, 47% for communication, and 39% for anxiety; improvement in stress and disruptive behavior: HSQ 29% and APSI 33%; adverse events included sleep disturbances 14%, irritability 9%, and loss of appetite 9%. Following the cannabis treatment, 33% received fewer medications or lower dosage, 24% stopped taking medications, and 8% received more medications or higher dose	Uncontrolled retrospective study of a subgroup of children with severe and refractory behavioral problems. Participants used various cannabis strains from different growers and a broad range of CBD and THC dose. The number of participants was not large enough to evaluate the impact on different ASD subgroups	Aran et al. (2019a)
*N* = 18 (72% M)/6–17 years old (mean 10.9 ± 3.06)	Observational study; cohort of 18 patients undergoing 6–9 months treatment with compassionate use of standardized CBD-enriched *Cannabis sativa* extract	CBD:THC ratio of 75:1 CBDRx^®^ (Colorado, USA), twice a day with an average CBD dose of 4.6 mg/kg/day and an average THC dose of 0.06 mg/kg/day. Starting CBD dose was ∼2.90 mg/kg/day (minimum: 2.30 and maximum: 3.60 mg/kg/day). Dosage adjustment occurred over 150 days. At the end of the study, the minimal CBD dose was 3.75 and the maximum was 6.45 mg/kg/day	Parents perceived percentage change on ADHD; BD; MD; AD; CSID; CD; sleep disorders; seizures. Clinical assessments: side effects and changes, maintenance, reduction, or withdrawal of neuropsychiatric drugs that were already in use	Retention rate in 6 months was 83% and in 9 months was 77%. Parents perceived percentage change: 47% had improvements equal to or above 30% in four or more symptoms categories, 13% presented improvements equal to or above 30% in two symptom categories, and 33% presented improvements equal to or above 30% in one symptom category. At least 60% of patients showed improvements of 20% or more in ADHD, MD, CSID, BD, sleep disorders, and seizures. Patients who presented BD: eight (53.3%) had improvements equal to or above 20% in this symptom category. AD, only four (26.7%) had improvements equal to or above 20%. ADHD, sleep disorders, and seizures, with more than 80% of patients presenting improvements equal to or above 30%. Five epileptic patients, with seizure reduction of 50% in three cases and 100% in the other two cases	Lack of control groups; small cohort size; potentially significant placebo effects due to caregivers bias. This treatment made it possible to achieve a decrease in the dosage or to discontinue other neuropsychiatric medications in eight out of 10 patients that were receiving OM	Fleury-Teixeira et al. (2019)
*N* = 53 (85% M)/4–22 years old (mean 11)	Prospective study; ASD children treated with CBD-oil over 30–588 days (∼1–19 months) had safety and comorbid symptoms assessed biweekly	CBD:THC ratio of 20:1 oil prepared by “Tikum Olam” at a concentration of 30%. Daily dose, maximal daily dose, and median interquartile range for CBD were 16 mg/kg, 600 mg, and 90 mg (45–143), respectively. Daily dose, maximal daily dose, and median interquartile range for THC were 0.8 mg/kg, 40 mg, and 7 mg (4–11)	According to parent’s reports, the emerging adverse effects, medications in use, and ASD comorbidities, hyperactivity symptoms, sleep problems, self-injury, and anxiety, were evaluated. An overall change was defined based on the summation of all parent’s reports. The change in each comorbid symptom in the study cohort was compared to published data using conventional treatment	Retention rate: 50% patients discontinued the treatment with 66 days. Overall improvement (*n* = 51) was reported in 74.5%, did not change in 21.6%, and worsened in 3.9%. Self-injury and rage attacks (n = 34) improved in 67.6% and worsened in 8.8%. Hyperactivity symptoms (n = 38) improved in 68.4%, did not change in 28.9%, and worsened in 2.6%. Sleep problems (*n* = 21) improved in 71.4% and worsened in 4.7%. Anxiety (*n* = 17) improved in 47.1% and worsened in 23.5%. Adverse effects were somnolence (*n* = 12) and decreased appetite (*n* = 6)	CBD shows noninferiority when compared to conventional treatments in the overall improvement of hyperactivity, self-injury, sleep problems, and anxiety symptoms	Barchel et al. (2018)
*N* = 188 (81% M)/5–18 years old (mean 12.9 ± 7)	Prospective study; children with ASD treated with medical cannabis (30% CBD and 1.5% THC) between 2015 and 2017	Cannabis oil with CBD:THC ratio of 20:1. The dosage ranged from 1 drop (0.05 ml) three times a day to 20 drops three times a day, for 6 months. Each drop (0.05 ml) contained 45% olive oil, 30% CBD (15 mg), and 1.5% THC (0.75 mg). The average dose was 79.5 ± 61.5 mg CBD and 4.0 ± 3.0 mg THC; patients with insomnia received an additional average dose of THC (3%) 5.0 ± 4.5 mg	Patient’s parents were interviewed and filled a medical questionnaire about demographics, comorbidities, habits, concomitant medications, measurements of quality of life, and a detailed symptom checklist. The evolution of patients was assessed after 1 and 6 months of treatment and intensity of symptoms, side effects, and quality of life were assessed. The global assessment approach and Likert scale were used to assess efficacy and quality of life, respectively	Quality of life (before the treatment): 31.3% of patients reported good quality of life, 3.3% reported good sleep, 0% reported good concentration, 42% reported positive mood, and 26.4% reported no difficulty in abilities to dress and shower independently. After one month, 179 patients (94.6%) continued treatment and 119 patients (66.4%) responded to the questionnaire. 48.7% reported a significant improvement, 31.1% reported a moderate improvement, 14.3% reported nonimprovement, and 5.9% reported side effects. After six months, 155 patients (86.6%) continued treatment and 93 patients (60%) responded to the questionnaire. 30.1% reported a significant improvement, 53.7% reported moderate improvement, 6.4% reported slight improvement, and 8.6% reported no change in their condition. 66.8% of patients reported good quality of life, 24.7% reported good sleep, 14% reported good concentration, 63.5% reported positive mood, and 42.9% reported no difficulty in abilities to dress and shower independently. 67 reported use of chronic medications, 8.9% reported an increase in their drug consumption, in 56.7%, drug consumption remained the same, and 34.3% reported a decrease. 23 patients discontinued the treatment and 17 (73.9%) responded to questionnaire for the treatment discontinuation: 70.6% reported no therapeutic effect and 29.4% reported side effects. Seven patients (41.2%) who discontinued the treatment had reported intentions to return to the treatment	The most prevalent side effect reported at six months was restlessness, appearing in less than 6.6% of patients. The compliance with the treatment was high and less than 5% have stopped the treatment due to the side effects. Absence of control group, therefore no causality between cannabis therapy and improvement in patient’s well-being can be established. Self-selection bias due to parents seeking cannabis therapy for their children. High compliance (above 80%) with the treatment provides good evidence of the patients and parents’ satisfaction with the treatment	Bar-Lev schleider et al. (2019)

Species/Strain/Sex	Study design/animal model	Dose and schedule of CBDV injections	Measurements	Main results	Comments	References
Preclinical study						
Rats/SD/M/	Single valproic acid administration (500 mg/kg; i.p.) on pregnant dams (GD 12.5) → ASD-like phenotype evaluated in their offspring	Daily injections of CBDV (0.2, 2, 20, or 100 mg/kg; i.p) from PND34 to PND58 (symptomatic protocol); daily injections of CBDV (2 or 20 mg/kg; i.p.) from PND19 to PND32 (preventive protocol)	*Behavioral assessment*: symptomatic treatment: three-chamber test on PND56, NOR (short-term memory) on PND57, and activity cage on PND58; preventive treatment: the same tests were performed on PND30, PND31, and PND32, respectively. *Molecular assessment*: 24 h after the last behavioral test in symptomatic protocol: expression of several proteins in PFC and Hp; immunohistochemical detection of Iba1 in dorsal Hp	Key behavioral effects: CBDV symptomatic treatment recovered social impairments, social novelty preference deficits, NOR deficits, repetitive behaviors, and hyperlocomotion; CBDV preventive treatment improved sociability and social novelty deficits, NOR impairments, and hyperlocomotion, without affecting stereotypies. Key molecular effects: prenatal VPA exposure increased CB1 receptor, FAAH, and MAGL levels, enhanced GFAP, CD11b, and TNFα levels, and triggered microglia activation restricted to the Hp. All these alterations were restored after CBDV treatment	CBDV increased CB2 receptor expression in Hp regardless of the gestational manipulation; both CBDV administration and prenatal VPA exposure decreased DAGLα expression in PFC	Zamberletti et al. (2019b)

AD, autonomy deficits; ADHD, attention-deficit/hyperactivity disorder; APSI, autism parenting Stress Index; ASD, autism spectrum disorder; BD, behavioral disorders; CBDV, cannabidivarin; CD, cognitive deficits; CGIC, caregiver global impression of change; CGI-I, clinical global impression of improvement; CSID, communication and social interaction deficits; F, female; GD, gestational day; Hp, hippocampus; HSQ-ASD, home situations questionnaire-autism spectrum disorder; M, male; MD, motor deficits; NOR, novel object recognition task; PFC, prefrontal cortex; PND, offspring’s postnatal day; SD, sprague-dawley; SL, sublingual; WB, western blotting.

### Clinical Evidence of Early Treatment with Products Containing CBD for ASD

The subjects in the clinical trials were ASD patients in distinct developmental stages (age range of 4–22 years) being a majority of boys. In all four studies, CBD was delivered as CBD-enriched cannabis extract oil containing both CBD and THC (and probably other cannabinoid molecules) administered orally. In three of them, the CBD/THC ratio was 20:1 (Barchel et al., 2018; Aran et al., 2019a; Bar-Lev Schleider et al., 2019), while in one study, it was 75:1 (Fleury-Teixeira et al., 2019). The treatments with CBD/THC oil presented elevated retention rates, achieving more than 80% retention after six months of treatment (Bar-Lev Schleider et al., 2019; Fleury-Teixeira et al., 2019), around 77% after nine months of treatment (Fleury-Teixeira et al., 2019) and 73% retention with a mean treatment duration of around 11 months (Aran et al., 2019a). On the other hand, in one study, the median retention rate was around two months (i.e., 50% of patients discontinued 1–2 months after starting treatment), ranging from one up to ∼19 months (Barchel et al., 2018). One can argue that lower retention rates in this study were due to the higher CBD dose used (16 mg/kg/day) when compared to lower doses in others with better retention rates (mean daily dose below 5 mg/kg; maximum dose of 10 mg/kg/day or less) (Aran et al., 2019a; Fleury-Teixeira et al., 2019). Since CBD dosage variation was broad in these studies, plus the fact that CBD-containing oil also contained other cannabinoids, an accurate conclusion about retention rates is difficult to be made. Notwithstanding, evidence regarding elevated adherence and retention rate for low doses of CBD in ASD patients is quite robust.

Around the reasons for discontinuation of CBD treatment, the most common were treatment ineffectiveness/low efficacy, the appearance of side effects, and a combination of both. Among the side effects reported, the most frequent were sleep disturbances, restlessness, sleepiness, irritability, and also loss or increase of appetite. It is essential to highlight that concomitant to CBD treatment, most patients were also receiving at least one of the following medications: typical or atypical antipsychotics, benzodiazepines or other anticonvulsants, selective serotonin reuptake inhibitors (SSRIs) or other antidepressants, stimulants, melatonin, etc. One can speculate that the adverse events observed throughout CBD treatment could be partially due to the synergic actions of other medications with CBD treatment. In fact, drug-drug interactions between CBD and lithium were reported in a 13-year-old boy with ASD and Lennox-Gastaut syndrome who presented lithium toxicity after a few weeks of treatment with 10 mg/kg/day CBD (Singh et al., 2020). In addition, since all the clinical trials reviewed here delivered CBD through oil extract containing THC and other compounds, the so-called "entourage effect" (i.e., a cannabinoid-cannabinoid interaction) cannot be ignored as a putative adverse effect cause (Cogan, 2020; Koltai and Namdar, 2020).

Even though some of the patients experienced adverse effects throughout treatment with CBD, improvements in ASD- and comorbidity-related symptoms were reported in all four studies. Immediate improvements in the patients’ behavior were observed, such as a decrease in anxiety, sleep problems, hyperactivity, rage attacks, and self-injury. Progress in the patients’ autonomy, increased motor, and cognitive performances as well as communication and social interaction improvements were also reported. The expected anticonvulsant effect of CBD (Mullard, 2018; Silvestro et al., 2019; Alves et al., 2020; Aran and Cayam-Rand, 2020; Lazarini-Lopes et al., 2020) was confirmed in two studies in which seizures were at least partially or even completely controlled (Bar-Lev Schleider et al., 2019; Fleury-Teixeira et al., 2019). In accordance with these studies, a recent case report about a 15-year-old boy with ASD who was treated with CBD-enriched cannabis extract oil (CBD/THC ratio of 20:1; 4 mg CBD and 0.2 mg THC twice a day) reported that CBD-based treatment aided in the control of ASD-related behavioral symptoms, core social communication abilities, anxiety, sleep difficulties, and body weight (Ponton et al., 2020). Notably, this study also reported that no side effects of the CBD-based treatment were observed. In addition to the direct impact that CBD treatment had on patients' behavior, parents and caregivers' indirect benefits were also reported. A decrease in patients’ disruptive behavior was observed and, consequently, improvements of 29% in the Home Situations Questionnaire-Autism Spectrum Disorder (HSQ-ASD) and of 33% in the Autism Parenting Stress Index (APSI) were reported (Aran et al., 2019a), indicating an increased quality of life for the whole family. A second indirect outcome regarded the concomitant use of other medications. Although few patients received more medications or higher doses after treatment with CBD, the proportion of patients who could reduce the dosage or even discontinue other medications was significantly higher (Aran et al., 2019a; Bar-Lev Schleider et al., 2019; Fleury-Teixeira et al., 2019).

The clinical evidence observed here suggests that early treatment with CBD might be a promising therapy for ASD. It yields important direct and indirect benefits (such as positive effects on multiple autistic symptoms and reduction in concomitant use of other medications). It also shows good tolerability without causing the typical side effects found in medicated ASD patients (in most cases, only mild and/or transient side effects were reported). However, it is essential to highlight the fact that methodological limitations were reported in all four studies. The two main self-reported limitations were due to 1) the unavailability of an objective assessment tool for symptom changes (the results were based on subjective reports of the patients’ parents or caregivers); 2) the nature of the studies: the lack of control groups could bias the outcomes, resulting in potentially significant placebo effects. Therefore, it is crucial that CBD's efficacy in treating ASD symptoms is confirmed through randomized, double-blind placebo-controlled multicenter trials. Fortunately, a clinical study (investigating both CBD and other phytocannabinoids) is currently being carried out (NCT03900923; NCT03849456; NCT03202303), although its results are not available yet. Additional studies must be conducted to better understand if CBD treatment benefits are indeed due to CBD effects *per se* or due to the entourage effect of cannabinoid molecules present in the cannabis oil extracts used in these studies.

### Preclinical Evidence of Early Treatment With Cannabinoids in ASD Models

Environmental manipulations during gestational periods have been used to induce an ASD-like phenotype in animals (Narita et al., 2002; Miyazaki et al., 2005; Schneider and Przewlocki, 2005; Narita et al., 2010; Malkova et al., 2012; Xuan and Hampson, 2014). These models focus on inducing at least some of the core ASD-like behaviors and/or neuroanatomical alterations in offspring. In rats, Zamberletti and colleagues (2019b) used the valproic acid (VPA) administration in the dams when they were in the 12th gestational day to induce an ASD-like phenotype. Their offspring were then treated with CBDV to investigate its effects on behavioral and molecular aspects related to ASD. As in VPA-exposed humans (Ornoy, 2009; Christensen et al., 2013; Veroniki et al., 2017; Macfarlane and Greenhalgh, 2018), VPA administration in pregnant rodents induced behavioral alterations in the offspring, including decreased social interaction, increased repetitive and stereotyped behaviors, hyperlocomotion, and impaired short-term recognition memory. In agreement with others (Schneider and Przewlocki, 2005; Servadio et al., 2016; Bronzuoli et al., 2018; Melancia et al., 2018), these behavioral alterations were observed in both the pubescent and early adulthood periods. The CBDV was administered in the offspring of VPA-treated dams (and in control ones) using two different therapeutic strategies. The first one was called the “symptomatic” approach in which several doses of CBDV (0.2, 2, 20, or 100 mg/kg/day) were tested: they were chronically administered throughout puberty (from PND34 to PND58) and the evaluations occurred at early adulthood (from PND56 to PND58). At this schedule, CBDV was efficient in reverting (or at least attenuating) all the VPA-induced behavioral abnormalities evaluated. The dose of 20 mg/kg/day was the most efficient one. The second CBDV therapeutic strategy was called “preventive”: CBDV (2 or 20 mg/kg/day) was chronically administered during an earlier period of neurodevelopment that encompassed a preweaning period plus the prepubertal period (from PND19 to PND32), and the evaluations occurred at puberty (from PND30 to PND32). Also, in this treatment schedule, the CBDV dose of 20 mg/kg/day was the most efficient. It reverted (or at least attenuated) the VPA-induced behavioral abnormalities evaluated, except for repetitive and stereotyped behaviors (measured through self-grooming).

Similar beneficial effects of chronic CBDV administration were observed in studies using genetic syndrome models, in which autistic behaviors are among the symptoms. Zamberletti et al. (2019a) found that chronic CBDV administration (at 20 mg/kg/day and others) in Mecp2 knockout mice (a Rett syndrome-like animal model) rescued the impaired short-term recognition memory which was evaluated during adolescence and early adulthood. In addition to CBDV benefits, chronic CBD administration (100 mg/kg twice daily, i.e., 200 mg/kg/day from the neonatal period up to early adulthood) rescued several autistic-like behaviors (anxiety- and depression-like behavior, poor social interaction, and increased rearing behavior, as well as reference memory and working memory) in *Scn1a* ^+/−^ mice, a Dravet syndrome-like animal model (Patra et al., 2020). Importantly, CBD did not induce any adverse effects on motor function, giving further support for the benefits and safety of using these cannabinoids in treating ASD.

As already discussed, the ECB system is altered in ASD patients and this might be directly related to the behavioral and morphological alterations observed in these individuals. This observation is also true for the animal models (for more information, see Zamberletti et al., 2017). Zamberletti and colleagues (2019b) found that CB_1_ and CB_2_ receptors' expression was increased in the hippocampus of VPA-treated animals. In addition, they observed that the expression of the two enzymes responsible for AEA and 2-AG degradation (FAAH and MAGL, respectively) was also increased in these animals while the expression of the enzymes responsible for the synthesis of these molecules (NAPE-PLD and DAGL-a, respectively) was not altered in the hippocampus. The CBDV symptomatic schedule treatment (i.e., chronic administration of CBDV from PND34 to PND58) rescued all of them except the increased CB_2_ receptor expression. The authors hypothesized that AEA and 2-AG concentrations are decreased in VPA animals (due to the increased expression of FAAH and MAGL) which agrees with other clinical and preclinical studies (Servadio et al., 2016; Karhson et al., 2018; Melancia et al., 2018; Wang et al., 2018; Aran et al., 2019b). They also suggest that the beneficial effects of CBDV could be related to the restoration of the ECB system abnormalities in the hippocampus. Contrary to the increase in ECB catabolic enzymes in the hippocampus, the DAGL-a expression was reduced in the PFC of VPA animals which agrees with the reduced 2-AG (but not AEA) hypothesis. However, the DAGL-a expression in PFC also decreased in response to CBDV treatment, which disagrees with the ECB system restoration hypothesis. A similar effect of CBDV was observed in cell culture experiments (De Petrocellis et al., 2011). In addition, reduced DAGL-a expression (related to decreased 2-AG levels) in response to chronic CBDV administration was also observed in the Rett syndrome model (Zamberletti et al., 2019a). In this case, administration of CBDV (at behaviorally effective doses) in the Mecp2 knockout mice increased the levels of AEA and oleylethanolamide (OEA, a monounsaturated analog of AEA that does not bind to cannabinoid receptors) while it reversed the increase in both CB_1_ and CB_2_ receptors. Interestingly, CBDV restored neurotrophic factor levels in Mecp2 knockout mice, which were related to a normalization of their common downstream AKT/mTOR signaling pathway and ribosomal protein six phosphorylation (Zamberletti et al., 2019a); both of them were expected to be impaired in ASD (Tai et al., 2020).

Substantial evidence suggests that immunological dysfunction plays a crucial role in the pathophysiology of ASD and that therapies able to control or reduce neuroinflammation could ameliorate ASD symptoms (Gottfried et al., 2015; Kern et al., 2015; Bjorklund et al., 2016; Bertolino et al., 2017; Bronzuoli et al., 2018). In the study by Zamberletti and colleagues (2019b), VPA injection during the gestational period induced hippocampal inflammation in the offspring, marked by enhanced levels of GFAP, CD11b, TNFα, and also microglial reactivity. The symptomatic schedule for chronic CBDV administration rescued both the hippocampal inflammation and autistic-like behavioral symptoms induced by gestational VPA injection, giving further support for this hypothesis. The anti-inflammatory actions of synthetic cannabinoids and phytocannabinoids have been extensively reported (Burstein, 2015; Schonhofen et al., 2018), especially for CBD and its derivative molecules. Some findings also support an anti-inflammatory property of CBDV (Tubaro et al., 2010; De Petrocellis et al., 2011; Amada et al., 2013; Pagano et al., 2019). On the other hand, chronic administration of this molecule induced an increase in GFAP expression in both control and VPA animals’ PFC (Zamberletti et al., 2019b), reinforcing the necessity for further investigation about this topic.

## Conclusion

Schizophrenia and ASD are psychiatric neurodevelopmental disorders that cause high levels of suffering, ranging from social isolation and cognitive deficits to severe debilitations and functional disabilities. The currently available treatments for these disorders are limited, stressing the importance of developing novel efficient and safe therapeutic strategies. The use of cannabinoids (as CBD and CBDV) during neurodevelopment (while the full-blown disorder symptoms are still in progress) has been investigated as a promising novel treatment for schizophrenia and ASD. However, the use of cannabinoid therapy demands particular caution since it must be safe both for the patients and for the individuals without a formal full-blown diagnosis. The clinical and preclinical evidence discussed in this review point out the beneficial potential that the treatment with CBD-based products (and/or CBDV for ASD) presents. Furthermore, the use of these cannabinoids was shown to be safe in both humans and animal models. Nevertheless, further clinical and preclinical studies should be carried out to provide more robust evidence for the use of CBD- (or CBDV) based products as an early preventive treatment for schizophrenia and ASD.

Even though the studies discussed here presented promising translational results, the number of studies investigating CBD (and/or CBDV) administration during neurodevelopment as a treatment for schizophrenia or ASD is still scarce. For schizophrenia, results from clinical studies investigating the effects of long-term treatment are not available yet. In addition, only ten preclinical studies investigating this issue have been published until now, limiting the complete translation of the data to clinical settings. The use of CBD for the treatment of ASD has been observed in four clinical trials, all of them using erratic CBD-enriched cannabis extract oils with other phytocannabinoid molecules (such as THC). In relation to preclinical trials, none using CBD during the neurodevelopment were performed and only one study using CBDV could be found. Another essential aspect that deserves attention is the ongoing lack of studies using female subjects, limiting the conclusions about the putative sexual dimorphism reported in the studies reviewed here. This issue is not restricted to preclinical investigations of psychiatric disorders, drawing attention to the fact that researchers should carefully plan their future studies to contemplate female subjects. Finally, the studies discussed in this review present an exploratory research approach. Therefore, their suggestive findings need to be further investigated through confirmatory research specifically designed to test the effect sizes identified in these studies as presenting biological relevance (Festing and Altman, 2002; Duan, 2013). Finally, further clinical long-term, placebo-controlled trials using pharmaceutical grade cannabinoids, involving different doses and neurodevelopmental treatment periods, would be timely to elucidate these compounds' potential in predicting better outcomes.

## Author Contributions

CL and VA were responsible for the conceptualization and design of the review. LT, GR, LM, and CL were responsible for reviewing the literature and acquiring the review data. CL, LT, FP, JC, AZ, JH, and VA were responsible for writing and revising the manuscript. All authors read and approved the final manuscript.

## Funding

This study was partially funded by Instituto Nacional de Ciência e Tecnologia Translacional em Medicina (INCT-TM), Conselho Nacional de Desenvolvimento Científico e Tecnológico (CNPq), and Fundação de Amparo à Pesquisa do Estado de São Paulo (FAPESP; 2008/09,009-2). The study was also funded by Coordenação de Aperfeiçoamento de Pessoal de Nível Superior (CAPES); JC received a grant from the University Global Partnership Network (UGPN), Global Priorities in Cannabinoid Research Excellence Program. VA, JC, JH, and AZ are recipients of CNPq research fellowships.

## Conflict of Interest

JC is a member of the International Advisory Board of the Australian Centre for Cannabinoid Clinical and Research Excellence (ACRE), National Health and Medical Research Council (NHMRC). JC and JH have received travel support to attend scientific meetings and personal consultation fees from BSPG-Pharm. JC, JH, and AZ are coinventors of the patent “Fluorinated CBD compounds, compositions and uses thereof. Pub. No.: WO/2014/108899. International Application No.: PCT/IL2014/050023,” Def. US number Reg. 62193296; July 29, 2015; INPI on August 19, 2015 (BR1120150164927; Mechoulam R, Zuardi AW, Kapczinski F, Hallak JEC, Guimarâes FS, Crippa JAS, Breuer A). Universidade de São Paulo (USP) has licensed this patent to Phytecs Pharm (USP Resolution No. 15.1.130002.1.1) and has an agreement with Prati-Donaduzzi to “develop a pharmaceutical product containing synthetic CBD and prove its safety and therapeutic efficacy in the treatment of epilepsy, schizophrenia, Parkinson’s disease, and anxiety disorders.” JC, JH, and AZ are coinventors of the patent “Cannabinoid-containing oral pharmaceutical composition, method for preparing and using same,” INPI on September 16, 2016 (BR 112018005423-2).

The remaining authors declare that the research was conducted in the absence of any commercial or financial relationships that could be construed as a potential conflict of interest.
